# Incidence of secondary rupture after abdominal aortic aneurysms (endovascular vs. open surgical repair)

**DOI:** 10.1016/j.amsu.2021.102831

**Published:** 2021-09-09

**Authors:** Ahmed Abdel Rahim, Rashid Ibrahim, Lu Yao, Ahmed Khalf, Mohammed Ismail

**Affiliations:** a plymouth University Hospital NHS Trust, UK; b Aswan University Hospitals, Egypt

**Keywords:** Infrarenal abdominal aortic aneurysm (AAA), Endovascular aneurysm repair (EVAR), Open surgical repair (OSR), Secondary rupture rate

## Abstract

A best evidence topic has been constructed using a described protocol. The three-part question addressed was: In patients with Infrarenal abdominal aortic aneurysm (AAA), Does endovascular abdominal aortic repair (EVAR), AS compared to open surgical repair (OSR), has lower secondary rupture rates? The outcomes assessed were the secondary rupture rate in both techniques. The best evidence showed that The OSR has statistically significant lower secondary rupture rates than the EVAR.

## Highlights

• Endovascular abdominal aortic aneurysm repair is inferior to the open surgical repair regarding the secondary rupture rates.
• Long term outcomes of the open surgical repair are better than the Endovascular repair.
• Secondary rupture is more common in endovascular repair than the open surgical repair of abdominal aortic aneurysm.

## 1 Introduction

This BET was designed using a framework outlined by the International Journal of Surgery [1]. This format was used because a preliminary literature search suggested that the available evidence is insufficient to perform a meaningful meta-analysis. A BET provides evidence-based answers to common clinical questions, using a systematic approach of reviewing the literature.

## 2 Clinical scenario

After resuscitating a 70-year-old man who presented to the emergency department (ED) with haemorrhaogic shock due to secondary rupture after open repair of infrarenal abdominal aortic aneurysm, one of the ED registrars asked; which modality of AAA repair has lower secondary rupture rates; EVAR or Open repair?

Three Parts Question:

• [In patients with AAA,]
• [Does EVAR has lower Secondary rupture rates];
• [As compared to OSR]?

## 3 Search strategy

A. Embase 1974 to June 2021 using the OVID interface: [AAA OR Abdominal Aortic Aneurysm]AND [Open repair OR open surgical repair OR OSR] AND [EVAR OR Endovascular Repair] AND [Secondary rupture]

B. Medline using the PubMed interface: [AAA OR Abdominal Aortic Aneurysm]AND [Open repair OR open surgical repair OR OSR] AND [EVAR OR Endovascular Repair] AND [secondary rupture]. The results were limited to English articles and human studies.

• Inclusion criteria: all original articles that review the secondary rupture rate among patients with AAA who underwent open surgical repair vs Endovascular Repair.
• Exclusion criteria: case reports, systematic reviews, letters to the editor, conference abstracts.

## 4 Search outcome

A total of 853 papers were found using both search engines. We excluded 812 papers because they were irrelevant based on the titles and abstracts. Forty-one full-text articles were screened and assessed for eligibility. From these, six papers were identified to provide the best evidence to answer the question.

## 5 Result

The search results are summarized in Table 1.

**Table 1:** Summary of search results.

Author/date of publication/journal/country	Study type and level of evidence	Patient group	Outcomes follow up	Key results	Additional comments
Lederle F A et al., 2019, N Eng J Med, USA [2]. OVER	Randomized control trial- Level 1b	Total of 881 patients with AAA * Group 1 EVAR: 444 *Group 2 OSR: 437 *Follow-up, 14.2 years. *Median was 9.4 years	*Endpoint is Secondary Ruptures. *Other outcomes: re-interventions, all cause and aneurysm related mortality	*Group 1 EVAR: 1.6% (7) patients. *Group 2 OSR: 0.2%% (1) patients. *95% confidence interval = 0.1–2.6. *P value = 0.03 *Statistically significant	*Long term *Multi Center *Specific skills and device training for the investigators.
Van Schaik et al., 2017, JVS, Netherland [3] Dream	Randomized controlled trial -level 1b	*Total of 351 patients with AAA *Group 1 EVAR: 173 *Group 2 OSR: 178 * Follow-up was 12 years. * Median was 10.2 years.	*Endpoint is Secondary Ruptures. *Other outcomes: re-interventions, all-cause, and aneurysm related mortality	*Group 1 EVAR: 4 (2.3%). *Group 2 OSR: 0 *P-value <0.05 *Statistically Significant	* Long term follow up *Multicenter *Lack of Blinding. *Old devices used
Rajesh Patel et al., 2016, Lancet, UK [4]. EVAR-1	Randomized control trial- Level 1b	*Total of 1252 patients with AAA *Group 1 EVAR: 626 *Group 2 OSR: 626 * Follow-up was 15·8 years. *Median was 12.4 years.	*Endpoint is Secondary Ruptures. *Other outcomes: re-interventions, all-cause, and aneurysm related mortality	*Group 1 EVAR: 31 (5%) Patients. *Group 2 OSR: 5 (0.8%) Patients. *95% confidence interval 2.50–16.75 *P-value < 0.05 *Statistically significant	* Long term *Multi Centre *Old devices used * Imaging was of low quality *Follow up changed from CT to Ultrasound .
Huang et al., 2015, JVS, USA [5].	Retrospective Cohort -Level 2a	*Total of 1116 patients with AAA *Group 1 OSR: 558 *Group 2 EVAR: 558 *Follow-up, 10 years; *Median was 7.6 years.	*Endpoint is Secondary Ruptures. *Other outcomes: re-interventions, all cause and aneurysm related mortality	Group 1 OSR: 0.17% (1) Patients Group 2 EVAR: 1.4% (8) Patients; * 95% confidence interval = ;1.01–64.99 *P value = 0.05 *Statistically significant	*Large sample size *Retrospective *OSR group are less rigorously followed up.
Kristina A Giles et al., 2011, JVS, USA [6].	Retrospective Cohort -Level 2a	Total of Patients with AAA *Group 1 EVAR: 22.826 *Group 2 OSR: 22.826 *Follow Up 6 years. *Median was	*Endpoint is re-interventions and Secondary Ruptures	*Group 1 EVAR: 441(19.3%) RR 0.50 *Group 2 OSR: 76(3.3%) RR 0.09 *95% Confidence Interval = 2.97–4.50 *P value < 0.001 * Statistically Significant	*Large Sample Size *Longitudinal design *Lack of clinical details

## 6 Discussion

Endovascular repair of abdominal aortic aneurysm repair has increased worldwide. It has become a powerful alternative to open surgical repair since Juan C. Parodi did the first successful endovascular repair in 1991 [7]. This is because of its minimally invasive nature and rapid recovery of patients. Recent good-quality randomized controlled trials reported better early outcomes and similar middle and long-term results to the open surgical repair [8].

In this article, we have reviewed the best studies which compared the open repair to the endovascular repair considering only the secondary rupture after the primary repair and any pre-interventional rupture were excluded.

All the five studies in our review reported statistically significant Lower secondary rupture rate difference in the OSR than the EVAR group. Three of them were Randomized controlled trials which were conducted by Lederle FA et al. [2], Van Schaik et al. [3], and Rajesh Patel et al. [4]. The other two were retrospective cohort trials and were conducted by Huang et al. [5] and Kristina A Giles et al. [6]. All of these studies included large sample size and long periods of follow-up.

## 7 Clinical bottom line

According to the above articles, the best evidence shows a statistically significant lower secondary rupture rate among patients with open surgical repair of abdominal aortic aneurysm in comparison to endovascular repair.

## Ethical approval

Ethical approval was not required.

## Sources of funding

No source of funding.

## Author contribution

Ahmed Abdel Rahim (AA): Conducted the literature search and wrote the paper.

Rashid Ibrahim (RI): Assisted in the literature search and Writing of paper.

Lu Yao(LY): Editing of writing.

Ahmed Khalf (AK): Assisted in writing of paper.

Mohammed Ismail (MI): Assisted in the literature search and writing of paper.

## Registration of research studies

1. Name of the registry:

2. Unique Identifying number or registration ID:

3. Hyperlink to your specific registration (must be publicly accessible and will be checked):

## Consent

Ethics committee approval was not required as the study was review of previously done studies.

## Guarantor

Ahmed Abdel Rahim.

## Declaration of competing interest

No conflicts of interest.
